# The muscle and neural architecture of *Taenia crassiceps* cysticerci revisited; implications on head-tail polarization of the larvae

**DOI:** 10.3389/fcimb.2024.1415162

**Published:** 2024-06-11

**Authors:** Arturo Calderón-Gallegos, Miguel Tapia-Rodríguez, Karel Estrada, Diana G. Rios-Valencia, Patricia de la Torre, Nicolás Castellanos-de Oteyza, Miguel A. Morales, Raúl J. Bobes, Juan P. Laclette

**Affiliations:** ^1^ Department of Immunology, Universidad Nacional Autónoma de México, Mexico, Mexico; ^2^ Unit for Massive Sequencing and Bioinformatics, Biotechnology Institute, Universidad Nacional Autónoma de México, Cuernavaca, Mexico; ^3^ Department of Microbiology and Parasitology, School of Medicine, Universidad Nacional Autónoma de México, Mexico, Mexico; ^4^ Department of Cell Biology and Phisiology, Biomedical Research Institute, Universidad Nacional Autónoma de México, Mexico, Mexico

**Keywords:** *Taenia crassiceps*, ORF, WFU, cysticercosis, muscle, nervous system, protonephridia, FMRF-amide

## Abstract

*Taenia crassiceps* has been used for decades as an experimental model for the study of human and porcine cysticercosis. Even though, its life cycle, tissue organization, ultrastructure and immune response elicited in the host, have been extensively described, there are many other biological questions remaining to be addressed. In the present study we revisited the muscle and neural architecture of cysticerci in two of the most frequently used strains (WFU and ORF), using conventional staining and confocal microscopy imaging, aiming to assemble an updated anatomy. Differences between both strains, including polarization processes during development of the young budding larvae, are emphasized. We also performed a search for genes that have been related to peptidergic neural processes in other related flatworms. These findings can help to understand the anatomical and molecular consequences of the scolex presence or absence in both strains.

## Introduction

Platyhelminths are invertebrate organisms including several taxa that have adopted a parasitic lifestyle (Campos et al., 1998; Littlewood et al., 1999). Among them, cestodes show an exquisite series of adaptations for parasitic life, including several species affecting humans and livestock (Tsai et al., 2013). *Taenia crassiceps* has been used as an animal model for the study of human and porcine cysticercosis caused by *Taenia solium*. Although, there is a close phylogenetic relationship between both species, anatomical, behavioral and physiological differences are apparent (Willms and Zurabian, 2010; Bobes et al., 2014). Three strains of *T. crassiceps* have been widely used: ORF (Ontario Research Foundation), WFU (Wake Forest University) and KBS (Kellogg Biological Station) (Freeman, 1962; Dorais and Esch, 1969; Fox et al., 1971; Sciutto et al., 2011; Reynoso-Ducoing et al., 2014; Rios-Valencia et al., 2022). A major difference between the three strains, is the lack of scolex in ORF, which has been proposed to be due to the loss of the second chromosome (Smith et al., 1972a). However, our recent characterization of WFU and ORF strains’ genomes, showed that the two genomes are almost identical, excluding the possibility of a chromosomal loss, and supporting another explanation involving differences in mechanisms of gene expression, so far unidentified (Bobes et al., 2022).

Cephalization has been regarded as an essential adaptive strategy in different types of platyhelminths (Halton and Gustafsson, 1996). In parasitic flatworms, such as cestodes, the head, known as scolex, has adopted another crucial role which is the anchoring of the worm to several host epithelia. Once attached, development of adult worm involves generation of new segments (strobilation) in the posterior part of the body (Bogitsh et al., 2019). Another feature of the scolex are the acetabula, also known as suckers, which are formed by finely innervated muscles (La-Rocca et al., 2019), neighboring cephalic ganglia which are organized in three-dimensional structures, connecting the four suckers through four fibrillar longitudinal cords running along the body of the tapeworm. The elongated and flat body plan of these parasites is also reflected in the distribution of nerve cords including lateral, dorsal, ventral and transversal nerves (Pax and Bennett, 1992; Halton and Maule, 2004).

Different molecular markers such as choline esterase (ChE), 5-Hidroxytriptamine (5-HT), gamma aminobutyric acid (GABA) and FMRF-amide related peptides (FaRPs) are present in the central or peripheral nervous system of cestodes (Fairweather et al., 1991; Halton et al., 1992; Vasantha et al., 1992; Sukhdeo and Sukhdeo, 1994; Koziol et al., 2013; Preza et al., 2018; Barčák et al., 2023). This has led to the description of the anatomy of the nervous systems in different cestode species. More recently, staining of these molecules with antibodies has revealed novel “neuro-exocrine” traits, including secretory glands releasing excretory/secretory (ES) products through the tegumental surface in contact with the host (Biserova et al., 2023a, Biserova et al., 2023b).

Even though many aspects of the basic biology of *T. crassiceps* have already been addressed, such as its reproduction and development, there is a lack of studies revising these processes through the use of novel methodologies. For example, the rate of cell division based on the incorporation of tritiated thymidine was assessed in early studies (Smith et al., 1972b), however, resolution can be improved through the use of other nucleotide analogues. Other unexplored issues are the presence or absence of a “central ganglion” in ORF, or the differential development and organization of the central nervous system in the anterior pole lacking a scolex. Herein, we have addressed these questions in order to update our knowledge of the cysticercus anatomy.

We describe the organization of muscle fibers, nervous and protonephridial systems in both strains of *T.* crassiceps (WFU and ORF), as well as the formation of an anlage which precedes development of the scolex in WFU involving a replicative cell population. Several genes associated with the nervous system were identified, which are present in both genomes, reinforcing our hypothesis that the absence of scolex in ORF is the result of a defect in developmental programming, instead of a result of a chromosomal loss (Bobes et al., 2022).

## Materials and Methods

### Parasite material

*Taenia crassiceps* cysticerci of the WFU and ORF strains were maintained via intraperitoneal transfer of cysts from infected to naive female BALB/c AnN mice 4 to 6 weeks old (Freeman, 1962). Infection proceeded for 90 days before humanitarian sacrifice of animals. Usually, 10 cysticerci were selected by size and injected into the peritoneal cavity of the recipient mice using 3 mL syringes with 21-gauge needles. Cysticerci were collected from the peritoneal cavity and washed in Phosphate Buffered Saline solution (PBS) pH 7.4 before use. All experiments with mice included in this study were approved by the Institutional Committee for the Care and Use of Laboratory Animals (CICUAL) at the Biomedical Research Institute, UNAM (permission No: ID 6329).

### Histological Procedures

*T. crassiceps* cysticerci, obtained as described above, were fixed in 4% formaldehyde during 12 hours for Masson trichrome (MT) staining. After fixation, cysticerci were dehydrated through graded ethanol solutions, followed by xylene, and subsequently embedded in paraffin. Tissue sections (5 µm) were obtained and placed on Superfrost Plus slides (Electron Microscopy Sciences, Hatfield, PA, USA). Following deparaffinization and rehydration, sections were MT stained following conventional methods previously described (Feldman and Wolfe, 2014; Van De Vlekkert et al., 2020). All sections were mounted with Entellan, examined and photographed under a light microscope (Nikon-Eclipse Ei with a Digital Sight 1000 camera).

### Proliferative cell tracking of intact cysticerci using 5-Ethynyl-2′-deoxyuridine EdU pulse-chase

Cysticerci were pulsed with 25 μM of EdU (Thermo-Fisher) in RPMI supplemented with 10% Fetal Bovine Serum (FBS) for 5 hours. Afterwards, cysts were washed with PBS and then placed in fresh RPMI, maintained at 37°C under 5% CO_2_ for 24 hours. Cysticerci were then rinsed with PBS and fixed in 4% formaldehyde in PBS for 30 minutes. After washing again, cyst tissues were permeabilized with fresh cold acetone for 5 minutes. EdU chase was performed according to the Click-It EdU (Thermo-Fisher) protocol. For nuclear staining, 4′,6-diamidino-2-phenylindole (DAPI) (1:500) was added. Finally, the resulting cysticerci were transferred to a 96 well plate and mounting solution was added (PBS-glycerol 40%).

### Whole-mount staining with phalloidin, FMRFamide or *Lens culinaris* agglutinin

Cysticerci were fixed in 4% formaldehyde in PBS for two hours. After fixation, larvae were rinsed three times for 10 minutes with PBS before proceeding on three different treatments: 1. For the visualization of the muscle architecture, cysticerci were permeabilized with acetone for 5 minutes and then thoroughly washed with PBS. The larvae were then placed in a solution of Flash Phalloidin green 488 (BioLegend 424201) at a 1:200 dilution in PBS for 30 minutes along with DAPI 1:500. After 3 more washes with PBS, cysticerci were mounted with PBS-Glycerol 40%; 2. For observation of the vesicular wall, larvae were placed in a PBS+SDS solution (1%) during 30 min to achieve permeabilization and washed three times with PBS+TritonX-100 (0.3%) (PBS-T). Blocking was carried out with PBS-T added with 3% BSA (PBS-T-BSA) for one hour at room temperature. Primary antibody against the conserved tetra-peptide FMRF-amide (Abcam ab10352) was added at a 1:1000 dilution in PBS-T-BSA, and the larvae were incubated overnight at 4°C. Afterwards, cysts were washed three times with PBS-T and the secondary antibody Alexa Fluor^®^ 488 AffiniPure Alpaca Anti-Rabbit IgG (H+L) (Jackson ImmunoResearch 611545215) at a 1:500 dilution in PBS-T-BSA and incubated overnight at 4°C. After three more washes with PBS-T, DAPI (1:500), phalloidin conjugated to Alexa Fluor^®^ 647 (Thermo-Fisher A22287) (1:500) and/or LCA coupled to DyLight 649 (Vector Laboratories, DL-1048–1) (1:500) were added and incubated for 30 minutes, before being washed again. Whole cysts were mounted in PBS-Glycerol 40% to prevent collapse of the fluid-filled bladder wall; 3. For observation of the scolex, we used proteinase K for permeabilization of the tissue as reported in *Echinococcus multilocularis* and *Hymenolepis diminuta* (Koziol et al., 2013; Rozario and Newmark, 2015). Cysticerci obtained from mice were placed in a PBS+SDS (0.5%) solution with proteinase K (2 μg/mL) for 5 min and then fixed again with 4% formaldehyde in PBS. Antibody, phalloidin and DAPI staining was performed as above.

### Neuropeptide precursor’s identification and differential gene expression analysis

Three hundred twenty-five sequences of neuropeptides have been annotated in twelve closely related species (*Clonorchis sinensis*; *Diphyllobothrium latum*; *Echinococcus granulosus*; *Echinococcus multilocularis*; *Gyrodactylus salaris*; *Hymenolepis microstoma*; *Mesocestoides corti*; *Opisthorchis viverrini*; *Protopolystoma xenopodis*; *Schistocephalus solidus*; *Schistosoma mansoni* and *Taenia solium*) (Koziol et al., 2016b). Based on previously identified genes reported by Bobes et al., 2022, we searched for neuropeptide genes in WFU and ORF *T. crassiceps* genome databases. The search was performed using the Blast (v2.14.1) program (Camacho et al., 2009). From this analysis, 30 orthologues were identified in the genotypes of both strains. Neuropeptides were manually curated and aligned using Clustal omega (Madeira et al, 2022).

### Differential Expression Analysis

We used Bowtie2 v2.3.4.3 (Langmead and Salzberg, 2012) to map the sequences from WFU and ORF RNAseq libraries to the coding sequences (CDS) of WFU strain. Subsequently, the counting matrices were obtained with Express v1.5.1 (Roberts et al., 2011) and finally the EdgeR v3.24.3 (Robinson et al., 2010) package was used to obtain the results of the differential expression analysis. To consider a gene as differentially expressed, a threshold of log_2_FC > 1 and < 1; as well as a false discovery rate (FDR) < 0.05 were used. All RNAseq results have been deposited in NCBI-Entrez and can be consulted under accession number PRJNA807072.

### Image acquisition

Confocal images were acquired at the Microscopy Core Facility (RRID: SCR_022204) using a Nikon A1R+ laser scanning confocal head, coupled to an Eclipse Ti-E inverted microscope (Nikon Corporation, Tokyo, Japan) equipped with a motorized stage (TI-S-E, Nikon) and controlled through Nis Elements C v.5.00 software. Cysts were analyzed under a Plan Fluor 10x DIC L N1 (N.A. 0.3) or PlanApo λ 20X (N.A. 0.75) objectives (Nikon Instruments); single plane images were sequentially captured using standard galvanometric scanners, excitation wavelengths of 405, 488, 561 and 640 with AOTF modulation, with variable pinhole apertures depending on the sample and fluorochromes utilized and both standard and GaAsP detectors.

## Results

### Proliferative cells localize primarily on growing buds

**Figure 1:**
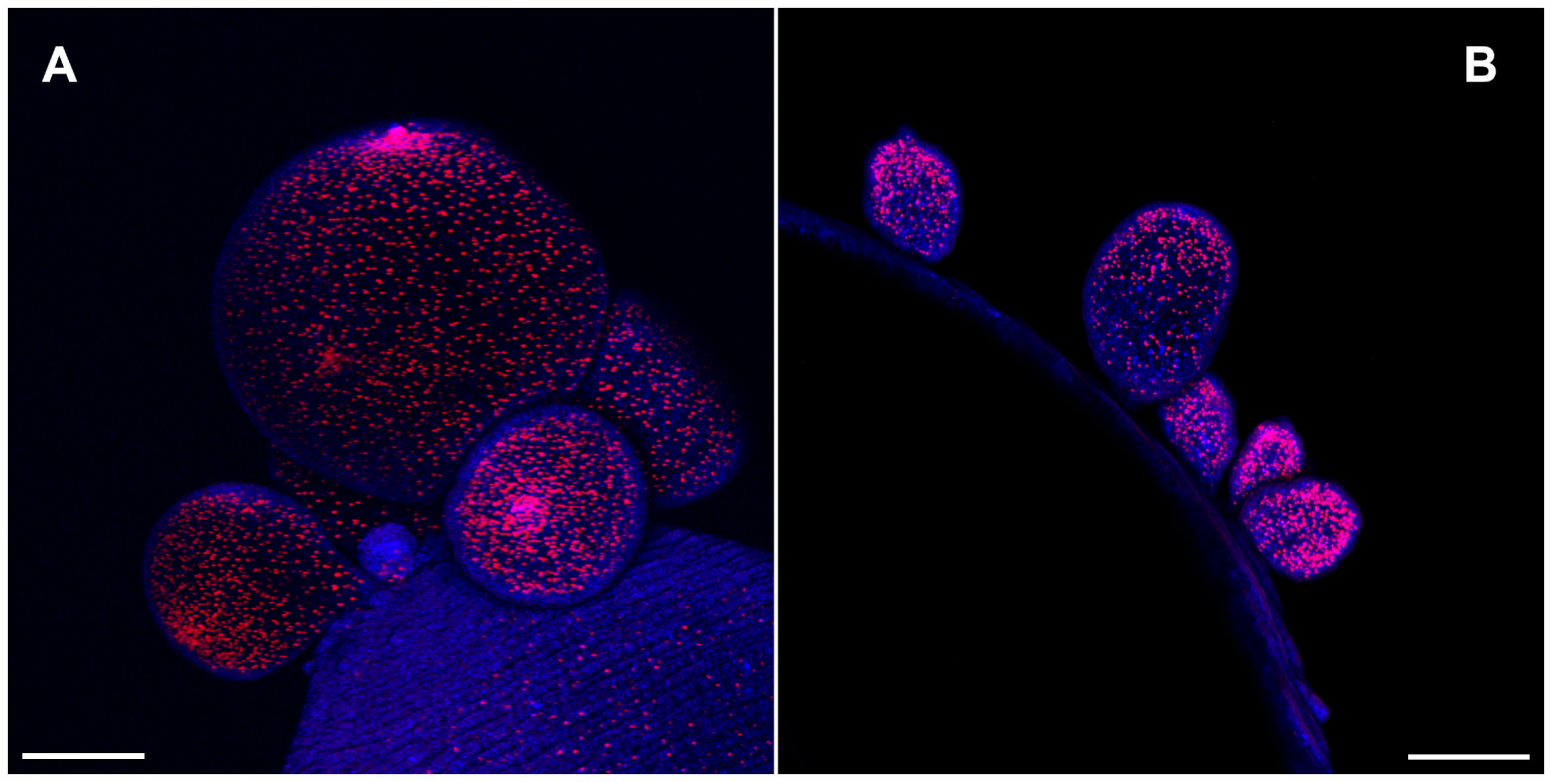
Cell proliferation visualized by 5-Ethynyl-2′-deoxyuridine (EdU) incorporation in the budding pole of *Taenia crassiceps* cysts. Whole-mounted cysts of WFU **(A)** or ORF **(B)** strains were stained with EdU (red) and DAPI (blue). Bar represents 200 μm.

*T. crassiceps* cysts reproduce by budding. Initial staining of cysts using EdU showed that proliferative cells were predominantly localized on the growing buds of WFU and ORF strains of *T. crassiceps* cysts (**Figure 1**). This is in agreement with previous observations evaluating DNA and RNA synthesis in developing buds of KBS and ORF (Smith et al., 1972b). In early developing cysts, it is often difficult to define anatomical references to guide morphological observations. Therefore, to establish a conventional anatomical plane, useful for comparisons between both strains, hereon we will designate the anterior end of the cyst, as the “scolex pole” and the posterior end as “budding pole”. This terminology is equivalent to the scolex and abscolex pole (Smith et al., 1972b), and aligns with recent studies carried out in *E. multilocularis* where the anteroposterior polarity due to the expression of different conserved markers between planarians and cestodes were described (Koziol et al., 2016a).

**Figure 2:**
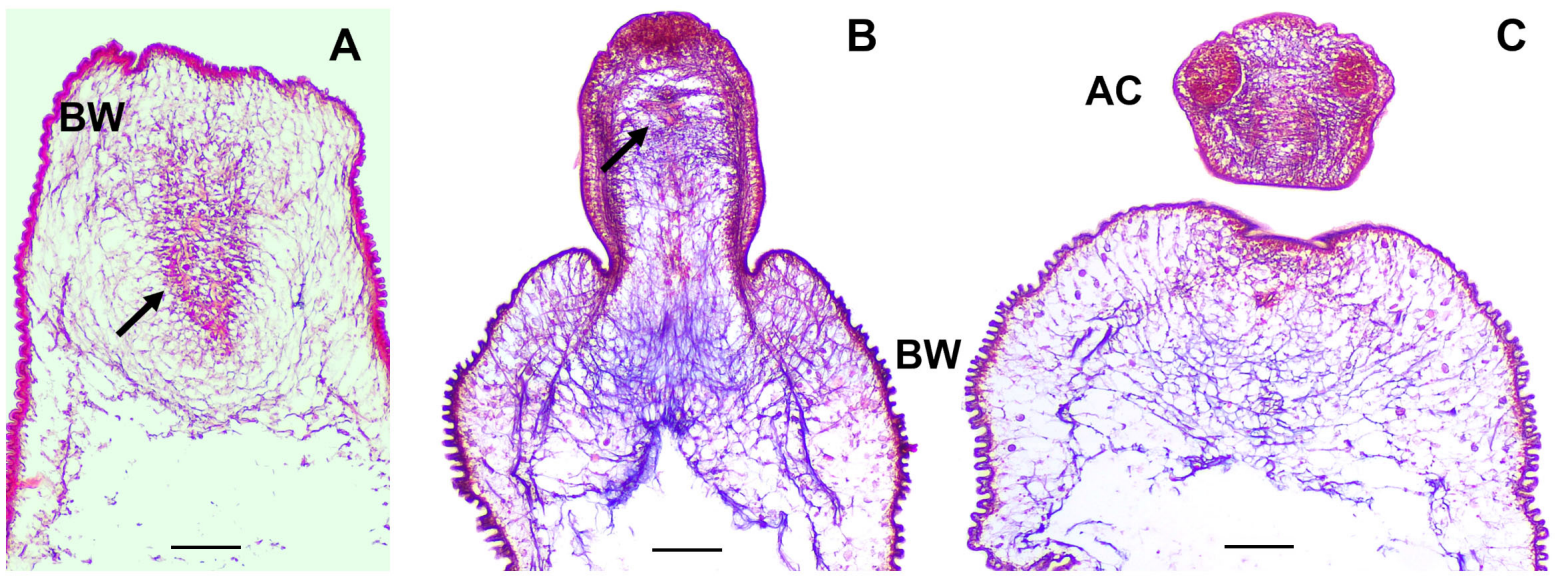
Masson’s trichrome staining on thick sections of *T. crassiceps* WFU and ORF strains. Arrows show the protonephridial system of the cyst. **(A)** ORF and **(B, C)** WFU cysts. Bladder wall (BW) and acetabula (AC). Bar represents 100 μm.

The scolex pole in the scolex lacking ORF strain is characterized by a slight engrossment of tissue as observed in MT staining of cysts sections (**Figure 2**) and contains protonephridial components such as flame cells and nephridial ducts, which are localized in the medullar region of this pole of the cysticercus. In contrast, the scolex pole in WFU is characterized by the presence of the prominent scolex structures, such as the acetabula, rostellum, nerve plexus and the cephalic ganglia. The protonephridial system was also visible in the scolex pole of WFU (**Figure 2**).

### Muscle architecture in the two strains of *Taenia crassiceps*

**Figure 3:**
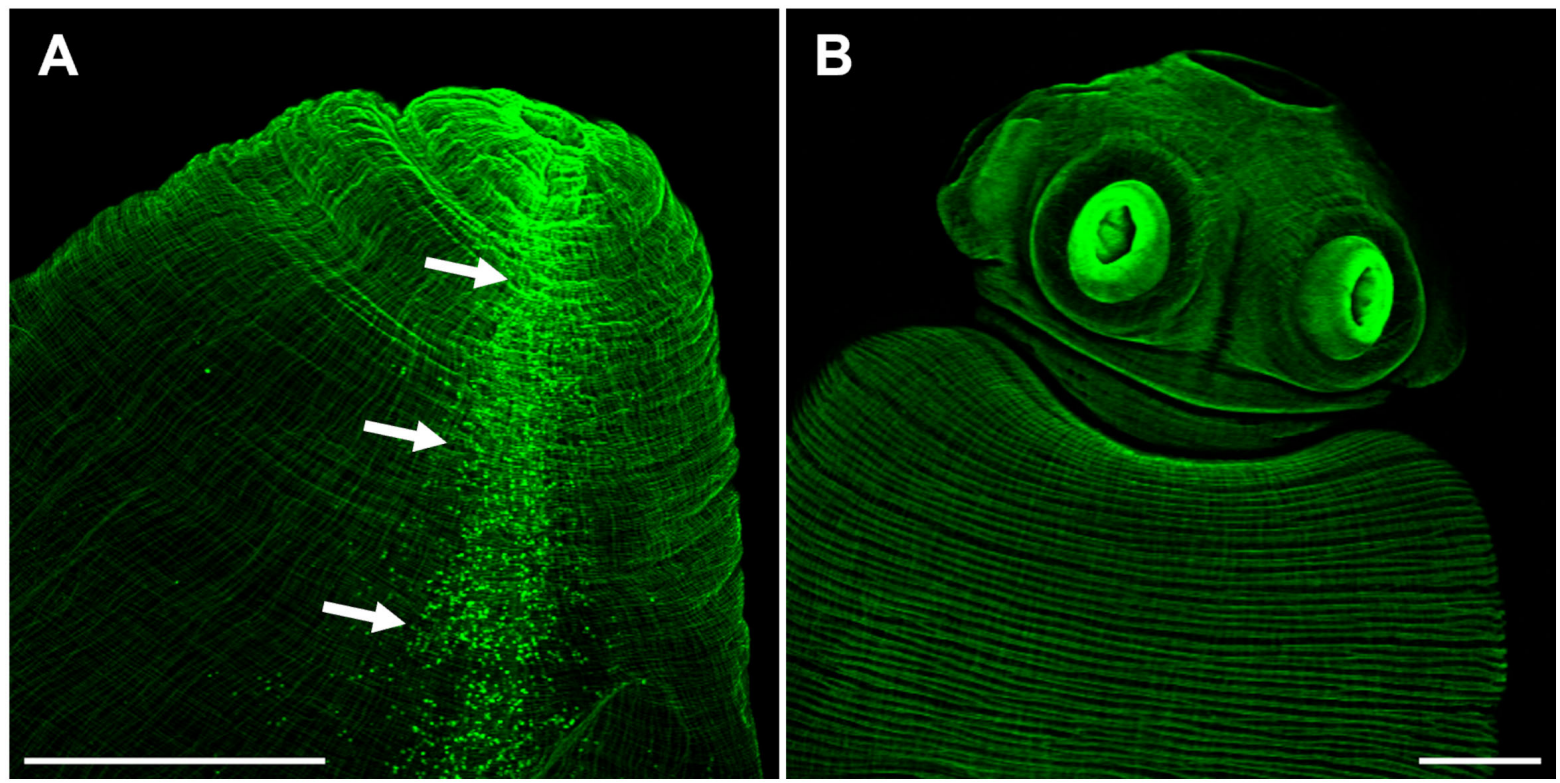
The scolex pole in *Taenia* crassiceps cysts. Whole mount phalloidin stained larvae from ORF **(A)** and WFU **(B)**. In A, arrows show numerous phalloidin positive cells (including flame and muscle cells) following the course of the lateral main cord. Bar represents 200 μm.

Phalloidin staining on whole-mount preparations of *T. crassiceps* cysts allowed visualization of a complex muscle fiber’s network (**Figure 3**). The series of confocal optical sections clearly showed the muscular architecture of the larva following a remarkably ordered arrangement of longitudinal (vertical) and circular (horizontal) muscle bundles. It is worth mentioning that such highly ordered musculature is responsible for the characteristic and continuous peristaltic-like contractions exhibited by the cysts under *in vitro* culture. The muscle architecture of the scolex could also be observed, because cysticerci usually evaginate spontaneously *in vitro* after 24 hours (**Figure 3**), and it resembles the organization described for other cestode species as *E. multilocularis*, *E granulosus*, or *Hymenolepis diminuta* (Koziol et al., 2013; Rozario and Newmark, 2015; La-Rocca et al., 2019). No phalloidin signal was detected inside the rostellar cavity (**Figure 3**). Abundant cell bodies were observed along the lateral main cords (**Figure 3A**), thus, phalloidin staining also allowed visualization of flame and muscle cell bodies. The rostellar cone, which is invaginated within the scolex, also presents a mesh of muscular tissue surrounding the hooks and the rostellar pad (**Supplementary Figure 1**).

**Figure 4:**
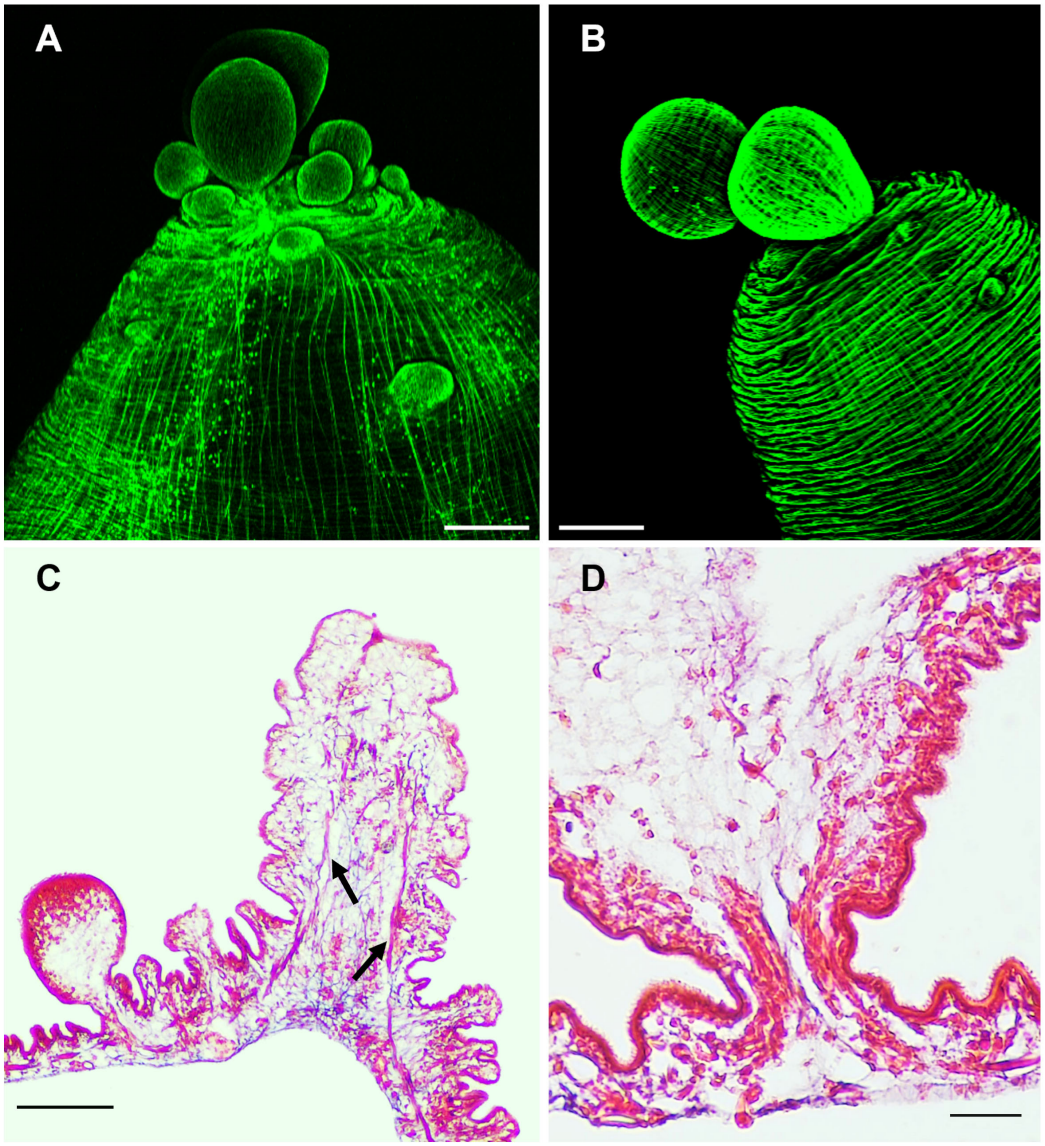
The budding pole in *Taenia crassiceps* cysts. Whole-mount Phalloidin stained ORF **(A)** and WFU **(B)** cysticerci. **(C)** Masson’s trichrome stained tissue from the budding pole of an ORF cysticercus. Arrows show the two lateral main cords. **(D)** Detail of an attached ORF bud in Masson’s-stained slides. Bar represents 200 μm in **(A, B)**, 100 μm in **(C)** and 20 μm in **(D)**.

The budding pole in both strains are similarly characterized by the presence of newly formed buds at different stages of development (**Figures 4A, B**). In new buds maintaining connection with the mother vesicle, muscles, nerves and protonephridia are present until final separation (**Figure 4D**). It has been proposed that the disassembly of the muscular, tegumental and protonephridial tissues precedes the final constriction before separation of new cysticerci (Bilqees, 1970). Interestingly, in the ORF strain, two prominent structures which end near the apical region of the budding pole stand out of the tissue sections, suggesting that these are the two main nerve cords of the larva (**Figure 4C**). As for the protonephridia, there are many observable duct-like structures and flame cells in the ORF strain which are not as prominent in the WFU cysticerci (**Supplementary Figure 2**).

### Identification of neuropeptide precursor genes and expression differences between ORF and WFU

For the specific staining of neural system, we decided to take advantage of the recently described neuropeptide precursors; expression patterns of neuropeptide precursor (npp) genes have been characterized in *E. multilocularis* and other parasitic flatworms (McVeigh et al., 2011; Koziol et al., 2016b). We carried out searches for orthologue sequences in the *T. crassiceps* genome databases including WFU and ORF strains. A total of 325 sequences belonging to 12 phylogenetically related species were analyzed, resulting in the identification of 30 *T. crassiceps* npp orthologues in both strains (**Supplementary File 1**). Moreover, based on previously reported RNAseq results (Bobes et al., 2022), expression levels of these newly described neuropeptide precursor genes and some of their corresponding receptors were compared. Changes in expression of npp orthologues in both strains are shown as the log_2_Fold Change (log_2_FC) in **Table 1**. As mentioned above, thresholds for up and downregulation of genes were established at log_2_FC <1 and >1, respectively. A total of 17 genes were found upregulated in WFU (log_2_FC <1), in contrast to only 2 upregulated in ORF (log_2_FC >1) (**Table 1**) (**Supplementary File 2**). Only one gene (npp 42) that was expressed in WFU was not expressed in ORF. These results suggested a widespread dysregulation in the expression of npp orthologues in ORF.

**Table 1:** Differences in the expression of neuropeptide precursors genes between *T. crassiceps* WFU and ORF strains.

Genes	log_2_FC	FDR
Upregulated in WFU		
npp 42	-10.3719	1.58E-24
npp 27	-3.0282	4.41E-06
npp 15.1	-2.5184	3.58E-08
npp 31	-2.5047	2.84E-22
npp 36	-2.4268	3.08E-14
npp 1	-2.1672	9.06E-09
Neuropeptide F	-2.044	9.20E-07
npp 29	-1.8996	5.22E-07
Neuropeptide prohormone-4	-1.8742	2.29E-13
npp 4	-1.8131	0.005844524
npp 20.5	-1.5699	1.22E-07
npp 40	-1.5225	0.000686118
npp 44	-1.3152	0.000333279
npp 14	-1.2081	0.012101342
npp 34	-1.1856	1.01E-05
npp 35	-1.1758	0.000661726
npp 20.2	-1.1206	0.040634488
Upregulated in ORF		
npp 20.3	1.2017	0.000238929
npp 24	1.7563	6.13E-12

The Log_2_FC of WFU expressed neuropeptide progenitors, is shown as a comparison with ORF.

### Neural architecture of WFU and ORF

**Figure 5:**
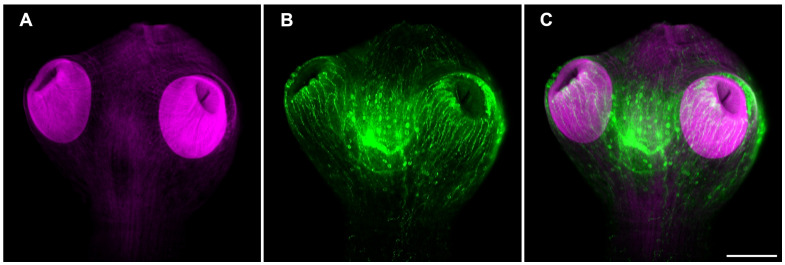
Muscle and neural architecture in the scolex of *T. crassiceps* WFU cysts. **(A)** Phalloidin staining of the scolex musculature (magenta). **(B)** FMRF immunoreactivity of a central ganglion and the suckers nerve plexus (green). **(C)** Merged images. Bar represents 100 μm.

The FMRFamide tetrapeptide is known to be present in a number of neuropeptides of different species of invertebrates (Richter et al., 2010; Krajniak, 2013). Having identified npp orthologues in *T. crassiceps*, specific staining using a polyclonal antiserum directed against FMRF related peptides precursor was done. The whole mount of WFU cysts showed an exquisitely specific staining of neural structures in combination with the formerly shown phalloidin staining (**Figure 5**). As shown above, acetabula showed an intense staining of the musculature with phalloidin. Neural structures showing FMRF IR appeared to wrap each sucker with a neural network at their base.

**Figure 6:**
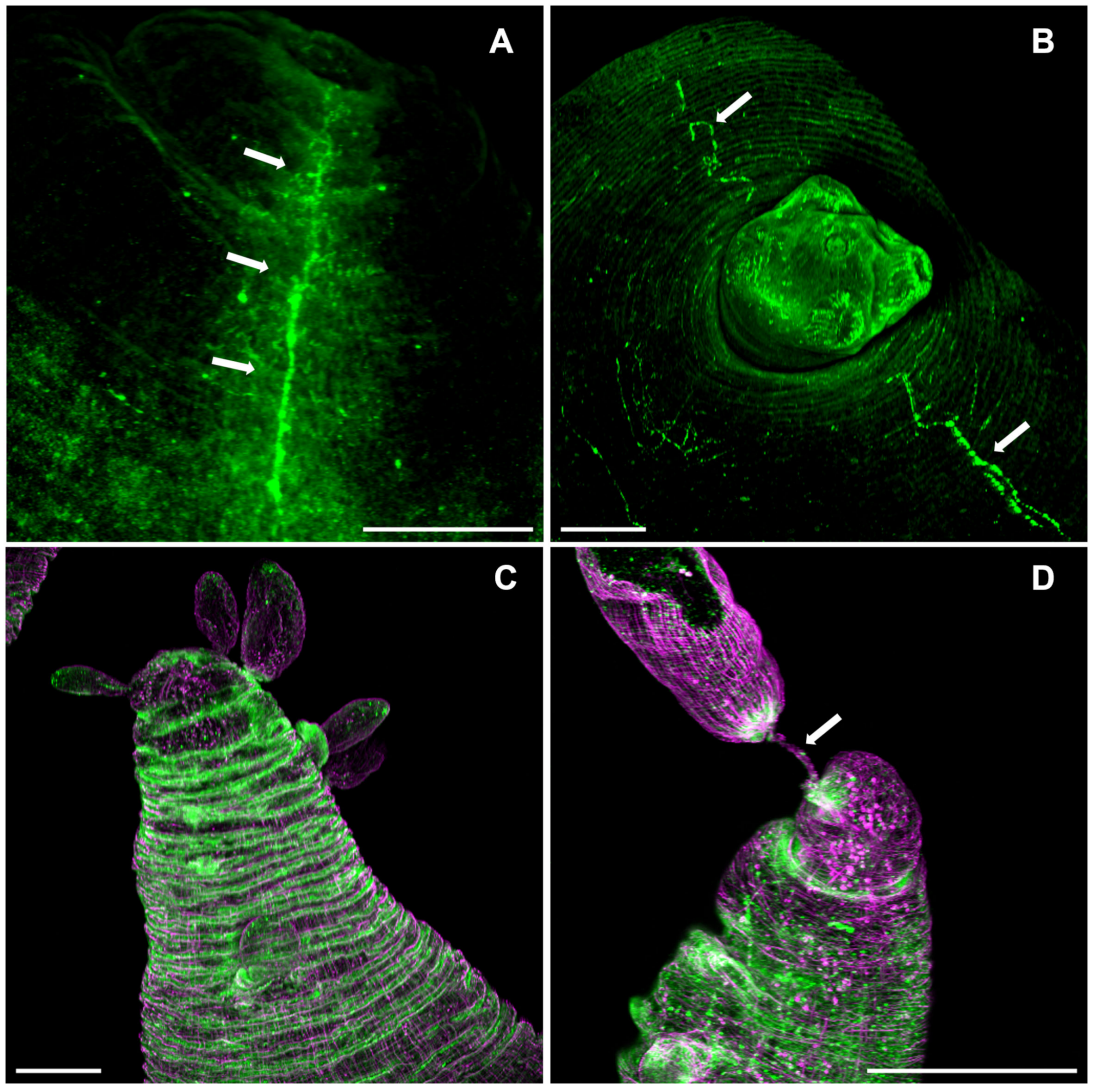
FMRF immunoreactivity pattern in *T. crassiceps* cysticerci. Arrows show the lateral nerve cords (green) in ORF **(A)** and WFU **(B)**. **(C)** Ring-like FMRF immunoreactivity pattern of the nervous system (green) and musculature (magenta) in the budding pole of an ORF cysticercus. **(D)** Muscular (magenta) and nerve (green) with the mother cyst (Arrow) in ORF. Bar represents 200 μm.

In the ORF strain, the pattern of FMRF IR was remarkably different; there was no clear regionalization of a central ganglion in the scolex pole (**Figure 6**). The only remaining nerves are the lateral main cords which run longitudinally along the cysticercus, such as the ones found in the WFU strain (**Figures 6A, B**). At the budding pole in ORF, a ring like pattern of FMFR IR was observed (**Figure 6C**). This distribution of neuropeptides was not seen in the budding pole of the WFU strain making clear the anatomical anomaly of this strain lacking scolex. Finally, the connection between the mother vesicle and the developing buds in ORF appeared similar to WFU (**Figure 6D**). Within the nascent cysticerci in ORF, there is a detectable staining pattern from FMRF IR and phalloidin. This is similar to what was observed in WFU (not shown).

### Polarization of early developing cysts

**Figure 7:**
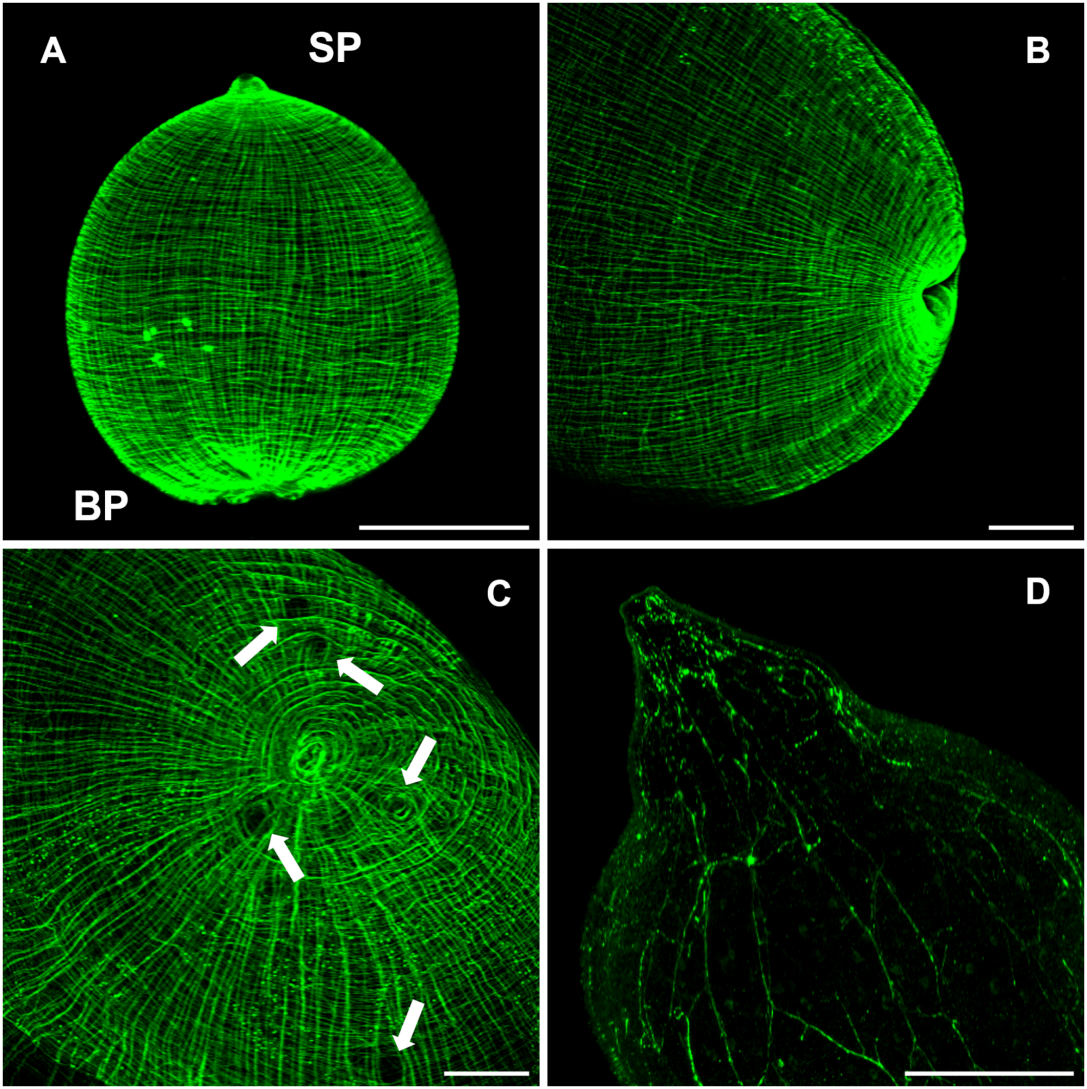
The budding process in WFU Taenia crassiceps cysts. Phalloidin staining **(A-C)** FMRF-IR in **(D)**. **(A)** Nascent cyst already showing polarization of structures. **(B)** Lateral view of the invagination of the tegument in the scolex pole. **(C)** Frontal view of the bladder wall constriction on the budding pole; arrows show black spots where new buds are forming. **(D)** FMRF immunoreactivity in the scolex pole. Scolex pole (SP) and budding pole (BP). Bar represents 100 μm.

The muscular architecture of the nascent buds stained with phalloidin, showed a clear polarization with the scolex and budding poles already identifiable (**Figure 7A**). On immature buds, the scolex pole showed an initial bump that precedes the invagination of the vesicle that will form the scolex (**Figure 7B**). On the opposite pole, the final constriction of the connecting “stalk” can still be observed, in the vesicle of nascent cysts. Regarding the budding process, a discontinuity within the circular and longitudinal mesh of muscle bundles could be observed, surrounded by the new budding sites (**Figure 7C**). From these spots, new cysticerci begin blebbing outwards from the mother cysticercus (**Figure 4**). Polarization is also observable in the early stages of cyst formation by the concentration of replicative cells visualized by EdU incorporation (**Figure 1**) on one pole of the nascent bud. A tip is indicative of the primordial scolex, moreover, this polarization is accompanied by formation of a neural network evidenced by FMRF IR (**Figure 7D**).

### The protonephridial system in *Taenia crassiceps*

**Figure 8:**
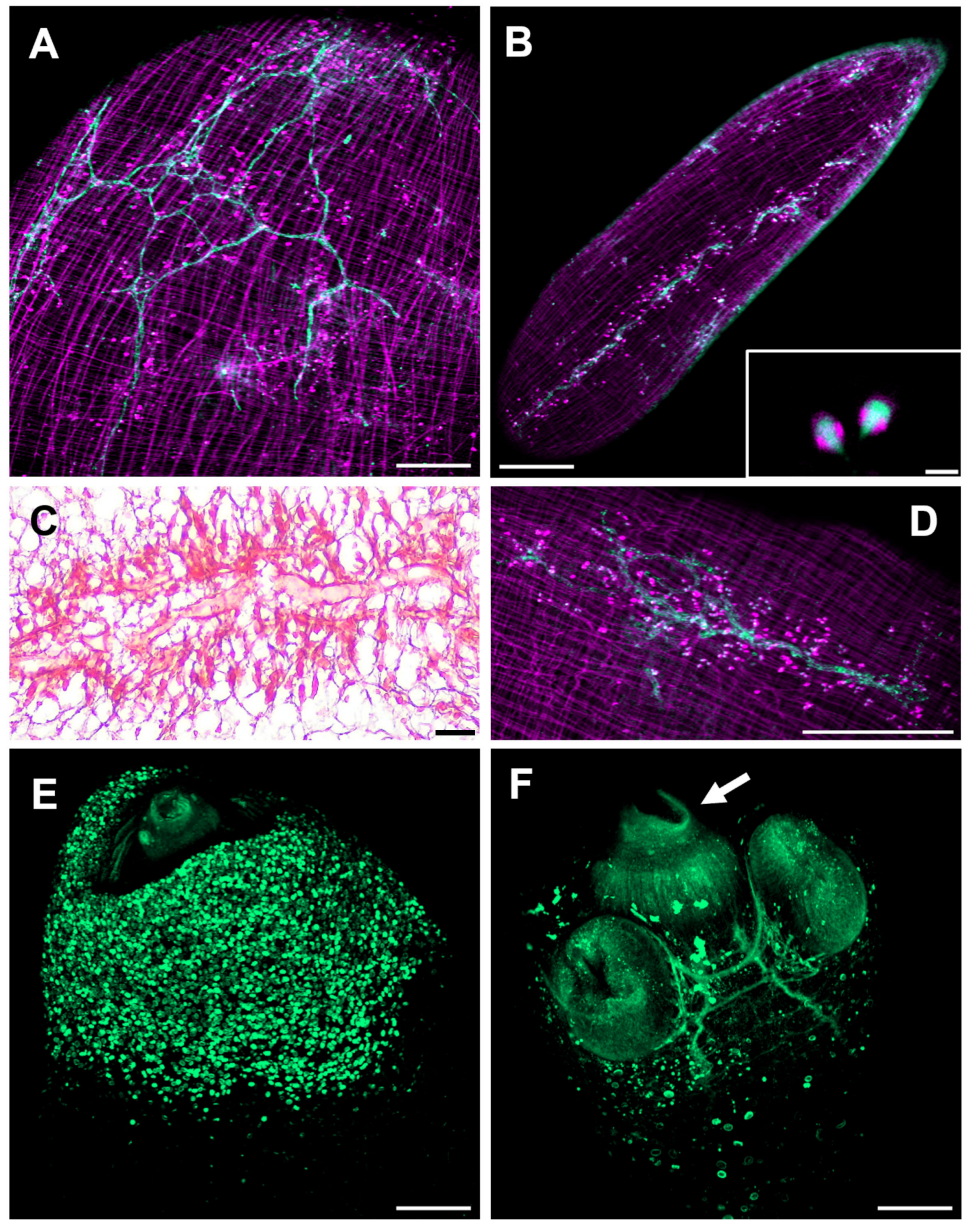
The cysticercus protonephridial system visualized by *Len culinaris* agglutinin (LCA) staining. **(A)** WFU and **(B)** ORF protonephridial systems stained by LCA (green) and phalloidin (magenta). Inset: Detail of classical flame cells **(C)** Masson’s trichrome staining of larval protonephridial ducts. **(D)** Anastomosing protonephridial ducts surrounded by flame cells. **(E)** LCA (green) stained calcareous corpuscles in the scolex pole of a WFU cysticercus. **(F)** Main protonephridial ducts in the scolex. Arrow shows LCA positive cells within the rostellar space. Bar represents 200 μm. Inset bar represents 5 μm.

In Masson’s trichrome stained tissue sections main protonephridial ducts were located in the cyst’s scolex pole of both strains (**Figures 2**, **8C**). In WFU, the network of protonephridial ducts was observed in the parenchyma of the scolex, whereas in ORF, possessing just a primordial parenchyma, a reduced network of ducts was also present. Flame cells were observed in close relationship with the protonephridial canals. To gather further detail of the protonephridial system of both strains, we used LCA, which has also been used for this purpose in *Hymenolepis diminuta* adult worms, Macrostomum lignano tissue labeling and to identify secretory cells in planaria (Zayas et al., 2010; Rozario and Newmark, 2015; Lengerer et al., 2016). Whole-mount visualization of *T. crassiceps* revealed protonephridia along the body of the larvae, constituted by an anastomosing network with a variable distribution of flame cells (**Figure 8D**), interconnected to larger collecting canals in WFU and ORF (**Figures 8A, B** respectively). Interestingly, the classical structure of flame cells (Valverde-Islas et al., 2011) was also revealed by LCA and phalloidin staining, allowing identification of flame cells (LCA and phalloidin positive) from muscle fibers (phalloidin positive only) (inset in **Figure 8B**). In this case, cell body of flame cells were delineated by phalloidin, whereas cilia appeared stained by LCA. Calcareous corpuscles were also prominently stained in both strains (**Figure 8E**).

Cysticerci of the WFU strain evaginated spontaneously after about one day under *in vitro* culture. In those evaginated cysts, the protonephridial system was readily visualized in the scolex parenchyma as an orthogonal arrangement with ducts connected from the base of suckers, along the neck (**Figure 8F**). LCA also stained groups of cells with no resemblance to other cell types lining the rostellar cavity in WFU. Identification of these cells deserves further investigation.

## Discussion

The basic morphology of cysts, including nascent buds of WFU and ORF strains of *T. crassiceps* was revisited using modern methodologies, to establish an anatomical reference. In addition to conventional staining like Masson’s trichrome we used EdU for localization of proliferative cells that were predominantly localized in growing buds of cysts; phalloidin was useful for visualization of a remarkably organized network of muscle fibers; FMRF-IR allowed certain identification of the neural anatomy of cysts, whereas LCA led to specific staining of the protonephridial system components. All these staining techniques provided the basis for the establishment of a detailed anatomical reference for cysts.

Through the comparison of RNAseq results between the wildtype WFU strain and the mutant ORF strain of *T. crassiceps*, our previous proposal suggested that the lack of scolex in ORF was not caused by a chromosomal loss occurring in the ancestor as proposed before (Smith et al., 1972a), but by a more complex and subtle gene dysregulation, resulting from epigenetic changes and/or mutations that remain unknown. Previously, we have shown that both genomes are almost identical making the identification of these changes an ulterior objective. One possible clue could reside in our findings related to neuropeptide expression in WFU and ORF. From the differential expression analysis, which identified 30 npp genes, 18 were dysregulated in the ORF strain; 17 were downregulated and two upregulated. Therefore, considerable changes in the expression of this group of genes are evident in the mutant strain. Similar to WFU and in contrast to ORF, several npp orthologues are expressed in *E. multilocularis* protoscolices (Koziol et al., 2016b), such as npp-27, npp-29, npp-31, npp-34, npp-36 and npp-42. However, further assessment of the main enzymes involved in the synthesis of neuropeptides along development of both strains of cysticerci is needed to elucidate the biochemical mechanisms participating in neural communication processes.

As previously shown, the expression of different neuropeptide genes can be visualized by FISH (Koziol et al., 2013; Koziol et al., 2016b; Preza et al., 2022; Montagne et al., 2023). Even though the specific location of these transcripts could be visualized in several body regions of the larvae or adult worms, its function throughout different developmental stages remains to be assessed with techniques such as RNAi. Our results contribute to understand the participation of the nervous system in the establishment of the larvae in the host. It is in this regard that identification of drugs targeting molecules involved in the nervous system of the parasite may become useful anthelmintics, especially against larval stages. Different markers of neurotransmitter systems have been shown to be present during key developmental stages, for example, prohormone convertase 2 (pc2), choline acetyltransferase (chat), vesicular glutamate transporter (vglut) and tryptophan hydroxylase (tph) in *Hymenolepis microstoma* (Montagne et al., 2023). Considering its role in larval development, different anthelmintic drugs specifically directed towards these components could be proposed. Another avenue for drug identification is the use of plant extracts with active principles possibly interfering with neural processes (Ukil et al., 2018).

Phalloidin staining allowed visualization of an exquisite and highly ordered muscle architecture in both strains of cysticerci, resembling parallels and meridians of the cyst. The highly motile *T. crassiceps* cysticercus (both strains) has a much more profuse arrangement of phalloidin stained muscle fibers than *E. multilocularis* (Koziol et al., 2013), also seen in Masson’s trichrome stained slides. This could be related to the need of an energic constriction of the stalk to allow separation of the nascent from the mother cyst. In contrast, the muscle architecture in the scolex of both species are more similar.

The nervous system of *T. crassiceps* could be revealed using FMRF IR; antibodies raised against the tetra-peptide have been used to reveal the anatomy of the nervous systems in several invertebrates, sharing this conserved sequence. FMRF IR showed different staining patterns in the two strains of *T. crassiceps* cysticerci evaluated here, being the lack of centralization of the nervous system in ORF, perhaps the most obvious anatomical difference between these strains. Our genomic and RNA expression analyses suggested that the differential expression of npp genes seems related to the lack of scolex, lodging a central ganglion in WFU, as well as in other cestodes (Halton and Maule, 2004). Both WFU and ORF cysts have lateral main cords, which run longitudinally in the vesicle to the budding pole. In ORF, this innervation was accompanied by a dense population of flame cells located near the nerve cord. The scolex in the WFU strain, was deeply innervated as revealed by FMRF IR and had a similar structure as seen in other related cestodes such as *T. solium, E. multilocularis* and *H. diminuta* (Vasantha et al., 1992; Sukhdeo and Sukhdeo, 1994; Koziol et al., 2013). The cephalic ganglia, which is the centralized structure of the nervous system, formed connections between the acetabula. This could be related to the need for muscular coordination during attachment of the evaginated scolex to the intestinal epithelium. Furthermore, the lateral main cords connected to the main ganglion allow communication with other tissues along the body wall of the cysticercus. Nonetheless, the lateral main cords in the ORF strain did not appear connected to any observable ganglion (**Figure 6A**).

Interestingly, FMRF IR in ORF cysts resulted in ring-like structures observed in the bud-forming pole that were not present in WFU. To our knowledge, this has not been reported before, including previous studies on *T. crassiceps* cysticerci using FMRF-amide staining (Fairweather et al., 1991; Koziol et al., 2013). It has been suggested that nervous and muscle cells are the source of several factors controlling the expression of positional control genes (PCGs) in flatworms (Koziol et al., 2016a). A dysregulated secretion of factors could explain reproductive and antigenic differences between WFU and ORF.

Another observation regarding nervous system components in the budding larvae, is that the connection with the “mother” cyst is retained until complete detachment of the new vesicle. It is conceivable the existence of a maturity “threshold” for the forming *T. crassiceps* cysticercus, before strangulating the stalk with the mother cyst. This appears not to be the case for *E. multilocularis* where the cyst nervous system does not seem to play an organizing role for the development of protoscolices during the budding process (Koziol et al., 2013).

Newly developed buds observed in WFU underwent a clear polarization. Phalloidin staining showed that buds organized their muscular system forming a stub-like end, from which the primordial scolex develops, in consistency with a pioneer description several decades ago (Chew, 1981). The nervous system develops gradually in the form of an orthogonal network, until separation of the mature cysticercus. EdU staining, evidencing cell proliferation was also seen in the scolex pole of the nascent buds, associated with scolex development (Bilqees and Freeman, 1969; Mount, 1970). The polarization process in ORF cysts, can be distinguished by the concentration of proliferating cells in the slightly engrossed scolex pole. After the bud’s development is complete, the stalk of the new larvae becomes constricted, and it has been proposed that the disassembly of different tissue components is needed to separate the larva (Bilqees, 1970). In the newly stablished budding pole of the forming cysticerci, the highly organized muscle mesh is gradually formed.

ORF cysts multiply much quicker than other strains possessing scolex (Dorais and Esch, 1969). Detoxification is restricted to the protonephridial system in the bladder wall, however, LCA staining revealed a similar anastomosing duct system near flame cells, when compared with WFU. Flame cells are known to promote circulation to the protonephridial liquid content, as each one of these cells are connected to a terminal duct where different components are excreted (Wilson and Webster, 1974; Valverde-Islas et al., 2011; Mustafina and Biserova, 2022). LCA staining also revealed flame cells cilia visualized by confocal microscopy (inset in **Figure 8B**). In *Hymenolepis diminuta*, LCA staining of adult worms only marked the cell body of flame cells and the collecting ducts (Rozario and Newmark, 2015). The rostellar surface also resulted stained by LCA, as previously shown for *H. diminuta* where the proposal of a new and unidentified cell type was advanced (Rozario and Newmark, 2015). Finally, calcareous corpuscles, which are considered final deposits of cyst’s chemical waste (Flores-Bautista et al., 2018), were also stained by LCA (**Figure 8F**).

## Conclusion

Characterization of different tissues and cell types in *Taenia crassiceps* cysts has been approached in an innovative manner thanks to recent methodologies implemented initially in other flatworms. Our confocal microscopy images have revealed the highly organized muscle, neural and protonephridial architecture throughout development of cysticerci. Our bioinformatic approach using NPP sequences aided to identify peptides potentially involved in a wide dysregulation of genes expression in the ORF strain. Herein, we describe the main aspects and differences observed in the nervous and muscular system of cysts. More specific molecular studies are needed in order to fully characterize differences between both widely used strains of *T. crassiceps*, to further elucidate the fundamental biology of taeniid cestodes.

## Data availability statement

The datasets presented in this study can be found in online repositories. The names of the repository/repositories and accession number(s) can be found in the article/**Supplementary Material**.

## Ethics statement

The animal study was approved by Consejo Institucional para el Cuidado y Uso de Animales de Laboratorio (CICUAL). The study was conducted in accordance with the local legislation and institutional requirements.

## Author contributions

AC-G: Conceptualization, Data curation, Formal analysis, Investigation, Methodology, Software, Visualization, Writing – original draft, Writing – review & editing. MT-R: Methodology, Software, Visualization, Writing – review & editing. KE: Data curation, Formal analysis, Methodology, Software, Writing – review & editing. DR-V: Data curation, Software, Visualization, Writing – review & editing. PT: Data curation, Formal analysis, Methodology, Software, Writing – review & editing. NC-DO: Methodology, Software, Visualization, Writing – review & editing. MM: Methodology, Resources, Visualization, Writing – review & editing. RB: Data curation, Formal analysis, Investigation, Methodology, Software, Visualization, Writing – review & editing. JL: Conceptualization, Data curation, Formal analysis, Funding acquisition, Investigation, Methodology, Project administration, Resources, Supervision, Visualization, Writing – original draft, Writing – review & editing.

## Supplementary material

Supplementary File 1 Predicted neuropeptide sequences of Taenia crassiceps WFU and ORF strains.

Supplementary File 2 RNAseq results of identified neuropeptide precursors.
