# Supplementary figures and images for: The muscle and neural architecture of Taenia crassiceps cysticerci revisited; implications on head-tail polarization of the larvae
Source: Front Cell Infect Microbiol. 2024 Jun 11;14:1415162. doi: 10.3389/fcimb.2024.1415162 (PMC11196405; doi:10.3389/fcimb.2024.1415162)

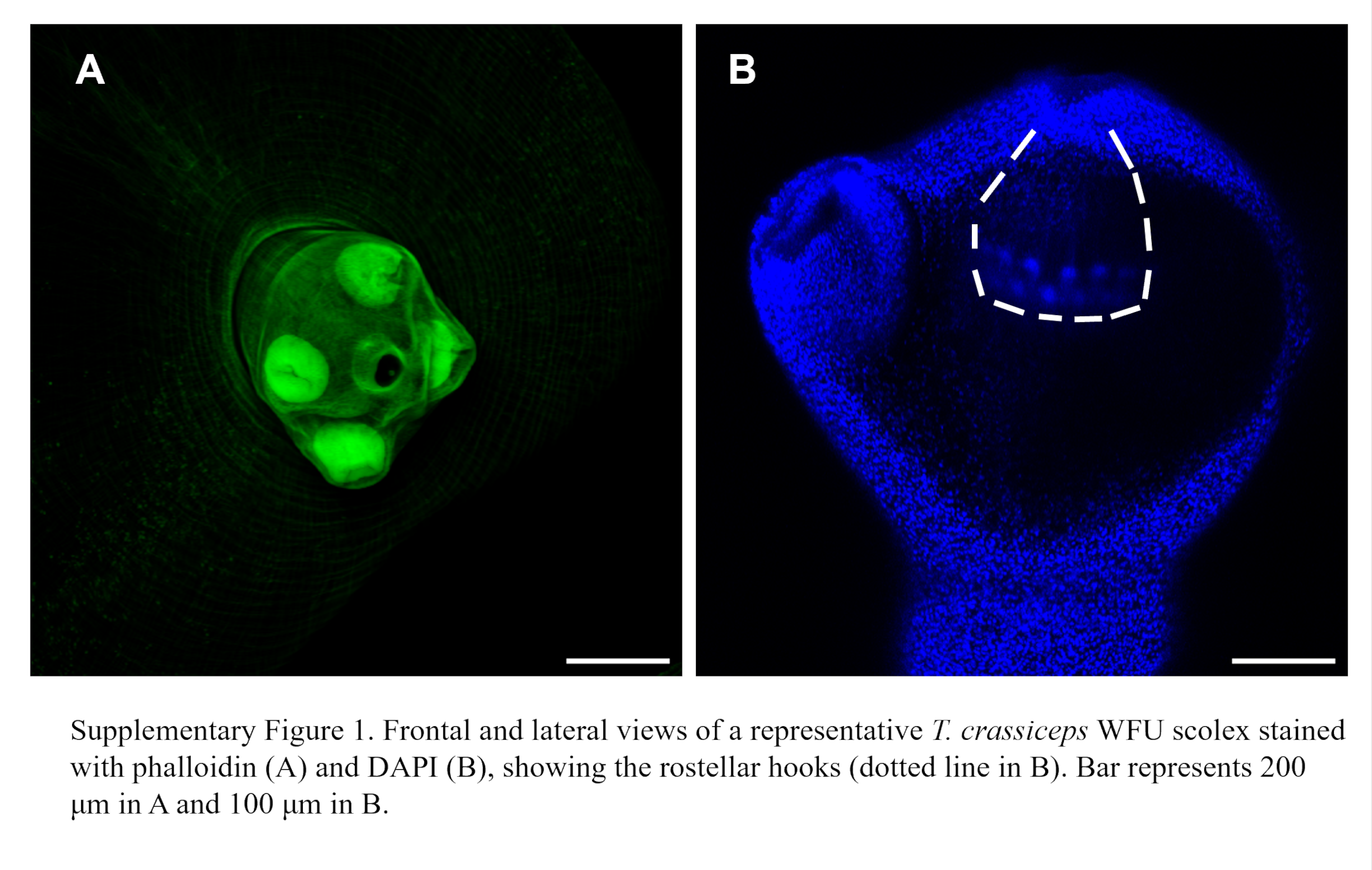

Supplement: Supplementary File 1 — Predicted neuropeptide sequences of Taenia crassiceps WFU and ORF strains. [file Image_1.tif]

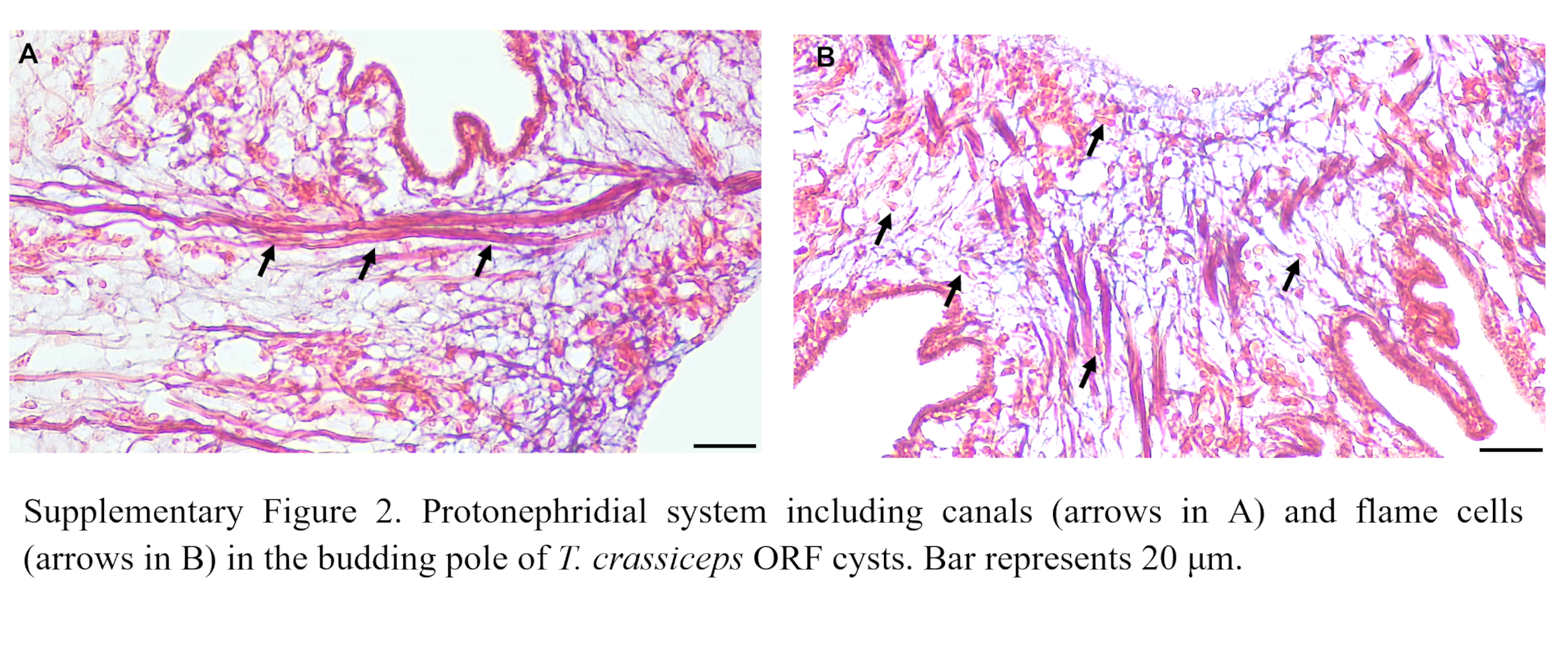

Supplement: Supplementary File 2 — RNAseq results of identified neuropeptide precursors. [file Image_2.tif]
