# Supplementary material for: The muscle and neural architecture of Taenia crassiceps cysticerci revisited; implications on head-tail polarization of the larvae
Source: Front Cell Infect Microbiol. 2024 Jun 11;14:1415162. doi: 10.3389/fcimb.2024.1415162 (PMC11196405; doi:10.3389/fcimb.2024.1415162)
Supplement: Supplementary file 3 [file Table_1.docx]

**Npp 1**

WFU TcrWFU_04493

MLTGNKLRAFMFTYKFAFCLSLVCFCLILLETPQTSGARIQRNLLGVLTNADPLEDPENQLFLEELNSLSPGLHRRLSSKRGFVRLG

ORF TcrORF_05281

MLTGNKLRAFMFTYKFAFCLSLVCFCLILLETPQTSGARIQRNLLGVLTNADPLEDPENQLFLEELNSLSPGLHRRLSSKRGFVRLG

**Npp 2**

WFU TcrWFU_07535

MVKQVCFVVFTAMIFYSQFSSATPLEGEDLVTLLVCRACEEAFPSSACLGSQEIQNTCLQSFLEEEDLFSQVSKRRGFIGKRDAGLQKRRGFIG

ORF TcrORF_06763

MVKQVCFVVFTAMIFYSQFSSATPLEGEDLVTLLVCRACEEAFPSSACLGSQEIQNTCLQSFLEEEDLFSQVSKRRGFIGKRDAGLQKRRGFIG

**Npp 4**

WFU TcrWFU_01963

MSYLRICFLCMFGIVMAFGMPVTRPDGAEGSLEGDPEDPESIDALQSRLEELFESLRQRRGNFFRFGKRSSPHRHRIDLLEKFLS

ORF TcrORF_09294

MSYLRICFLCMFGIVMAFGMPVTRPDGAEGSLEGDPEDPESIDALQSRLEELFESLRQRRGNFFRFGKRSSPHRHRIDLLEKFLS

**Npp 5 (trimmed)**

WFU TcrWFU_08911

MIVRASSTVFRLHLLLLVCLISELLTVGLAVEIHRTPTKRMQRPAFLFDAWGKRSAPQRPLWSLVGEDEDSYLVVIPKRGYKSVATELEDETENLYGPRQAAFKRGAYLDLPWG

ORF TcrORF_01838

MIVRASSTVFRLHLLLLVCLISELLTVGLAVEIHRTPTKRMQRPAFLFDAWGKRSAPQRPLWSLVGEDEDSYLVVIPKRGYKSVATELEDETENLYGPRQAAFKRGAYLDLPWG

**Npp6**

WFU TcrWFU_03740

MFPQCVDMKTAFAAITLMLLLTDPVIEALPSGPRGLERVDTDLELPPLAIEQVVDEKLSLVKDFLDDYFADQKGPSSTRKRAIRLLRLGK

ORF TcrORF_04581

MFPQCVDMKTAFAAITLMLLLTDPVIEALPSGPRGLERVDTDLELPPLAIEQVVDEKLSLVKDFLDDYFADQKGPSSTRKRAIRLLRLGK

**Npp 14**

WFU TcrWFU_00041

MSTYWQYLCLFTFLLIVRSSAARLAREGVDSGEFQLGDIGGELYKRGIMKMRIGKRGSDSGYEDFADEPESAYVYVPDGVMRAALKRAGIRRMRMG

ORF TcrORF_02442

MSTYWQYLCLFTFLLIVRSSAARLAREGVDSGEFQLGDIGGELYKRGIMKMRIGKRGSDSGYEDFADEPESAYVYVPDGVMRAALKRAGIRRMRMG

**Npp 15.1**

WFU TcrWFU_08484

MNKWTALTLFSVMVICLATMPSSQASSLGGHGSRGGVSEAAVDVASAVEFEALLQDYLEQMRDLKELIQRYLVSAPYSLSKRAQFLRLGRK

ORF TcrORF_02267

MNKWTALTLFSVMVICLATMPSSQASSLGGHGSRGGVSEAAVDVASAVEFEALLQDYLEQMRDLKELIQRYLVSAPYSLSKRAQFLRLGRK

**Npp 15.2 (trimmed)**

WFU TcrWFU_08483

MTKLRLCAQSWLALVYLLGYLTLPIGGMTINRFLGENEELVPGETYQYLPSIHPERVVMYDPMSGHVRSVWRYPAPPMSVIEQDSLPTVKRAQFLRIG

ORF TcrORF_02268

MTKLRLCAQSWLALVYLLGYLTLPIGGMTINRFLGENEELVPGETYQYLPSIHPERVVMYDPMSGHVRSVWRYPAPPMSVIEQDSLPTVKRAQFLRIG

**Npp 20.1 (Neuropeptide F)**

WFU TcrWFU_09602

MFAFVKSTPIYLVVLLALMVPLNTAGVHVDERRLARAANALRRQLEQQQQNQLQYRSPLSHSWLDVPLDVLDDGLTSSDYVKRTDPLSIVNPPESVLNNPAALRDYLRQVNEYFAIIGRPRFG

ORF TcrORF_01137

MFAFVKSTPIYLVVLLALMVPLNTAGVHVDERRLARAANALRRQLEQQQQNQLQYRSPLSHSWLDVPLDVLDDGLTSSDYVKRTDPLSIVNPPESVLNNPAALRDYLRQVNEYFAIIGRPRFG

**Npp 20.2**

WFU TcrWFU_02956

MQSTGSVTTMSPPLSVLGARADQDGPIDPQIVHWLQSQDPSLTTILNSNGNANYGNNSHSWKDLFKYMAQLNDYYVLFGRARFG

ORF TcrORF_06664

MMQSTGSVTTMSPPLSVLGARADQDGPIDPQIVHWLQSQDPSLTTILNSNGNANYGNNSHSWKDLFKYMAQLNDYYVLFGRARFG

**Npp 20.3**

WFU TcrWFU_05604

MVRKVTLAGIPTRPISWFGLVCLIVIVLSFVSKSEALFARSSVEEILPSKSGLTESRKQIFRNVKEFRRYLQRLDEWLAITGRPRFG

ORF TcrORF_03642

MVRKVTLAGIPTRPISWFGLVCLIVIVLSFVSKSEALFARSSVEEILPSKSGLTESRKQIFRNVKEFRRYLQRLDEWLAITGRPRFG

**Npp 20.4 (trimmed)**

WFU TcrWFU_00419

MSLMTISPLLITTLFLGSLQVSSTYHLPTSRFQDRFVPIERDSFPPYWMDDDDIDGEAEEVEVAGGRVDKRSRSTVPSLPQYLERLKKFHSLRDKEKYVAALNSYYMIFGRPSWS

ORF TcrORF_03560

MSLMTISPLLITTLFLGSLQVSSTYHLPTSRFQDRFVPIERDSFPPYWMDDDDIDGEAEEVEVAGGRVDKRSRSTVPSLPQYLERLKKFHSLRDKEKYVAALNSYYMIFGRPSWS

**Npp 20.5 (trimmed)**

WFU TcrWFU_05855

MRAPSTFHVCLLCVLLIFFQIHSVQGELERTRHSFDAFPLEYRERVKSVGPSIEQKRGTPAGGWDLADDLGTLEAGEDFGDINDAVLSSEKTSLTREEIQRYLAQMKAYYLKNGMPRYG

ORF TcrORF_07690

MRAPSTFHVCLLCVLLIFFQIHSVQGELERTRHSFDAFPLEYRERVKSVGPSIEQKRGTPAGGWDLADDLGTLEAGEDFGDINDAVLSSEKTSLTREEIQRYLAQMKAYYLKNGMPRYG

**Npp 24 (trimmed)**

WFU TcrWFU_09391

MFATLLLLSCLAMDSWSFQVDQSIPQTMALTDADLDVMEGPQLLKWPKRRLDKPAFYIPDDDVPTYLSGLRYLRSAQKRFKPKMPIFLGLIGKRK

ORF TcrORF_01356

MFATLLLLSCLAMDSWSFQVDQSIPQTMALTDADLDVMEGPQLLKWPKRRLDKPAFYIPDDDVPTYLSGLRYLRSAQKRFKPKMPIFLGLIGKRK

**Npp 26**

WFU TcrWFU_01956

MRAMLAVLFCALSFFACVSLAHPLSDEESLELFKRYFDPIRFAMGPIRTKEDLEKREAVVASKRYFDPILFKHAF

ORF TcrORF_09287

MRAMLAVLFCALSFFACVSLAHPLSDEESLELFKRYFDPIRFAMGPIRTKEDLEKREAVVASKRYFDPILFKHAF

**Npp 27**

WFU TcrWFU_00804

MLSISVHNRAIGGLPLFLLICMSLSPSTNAAAVGHDGSDSIKRRLPYLIGGIRYKRPLEPPPFPLSEVAAEEAVEAEELERLREALEASEEGRDLVKRALDYLYGGVRYRRRSVA

ORF TcrORF_00777

MLSISVHNRAIGGLPLFLLICMSLSPSTNAAAVGHDGSDSIKRRLPYLIGGIRYKRPLEPPPFPLSEVAAEEAVEAEELERLREALEASEEGRDLVKRALDYLYGGVRYRRRSVA

**Npp 29**

WFU TcrWFU_01870

MKAFCAVLFTVLVVIALTVAAEEPKSHEEEKRMLYWKRGGLEDLDDEELGTYDTLELNDSSLHNKANNFTELHLFIQTLSTYLGVPKFNRINAFAVLAHYQQTSLPYFHLQP

ORF TcrORF_09203

MKAFCAVLFTVLVVIALTVAAEEPKSHEEEKRMLYWKRGGLEDLDDEELGTYDTLELNDSSLHNKANNFTELHLFIQTLSTYLGVPKFNRINAFAVLAHYQQTSLPYFHLQP

**Npp 30**

WFU TcrWFU_06536

MGTSARPIVGMQRNQYNPSLPPHKRFQQNSPVLGCDLVVYRSTTSMILTHRVPAVRTQRGLLVYATAAMVFLIQTPACARHNALEEELALEDLPLSENIPDEDYFGVLPYRLDRRGFLHSGRLGRRGLLPSHQSSKRGFLTASRLG

ORF TcrORF_08342

MGTSARPIVGMQRNQYNPSLPPHKRFQQNSPVLGCDLVVYRSTTSMILTHRVPAVRTQRGLLVYATAAMVFLIQTPACARHNALEEELALEDLPLSENIPDEDYFGVLPYRLDRRGFLHSGRLGRRGLLPSHQSSKRGFLTASRLG

**Npp 31 / 32.2**

WFU TcrWFU_06776

MSSSSCCLLLLSALLLLSSFTEHSHIEARYLFVPKRVYFTSPSASALNLVNRHGAGSGGAVQGMSDQEDLLYPAPRLSQHMNKRGPEIFIPLMEA

ORF TcrORF_06453

MSSSSCCLLLLSALLLLSSFTEHSHIEARYLFVPKRVYFTSPSASALNLVNRHGAGSGGAVQGMSDQEDLLYPAPRLSQHMNKRGPEIFIPLMEA

**Npp 33 (trimmed)**

WFU TcrWFU_04423

MSPSHLLDLALLLTAFAAVDSLKVKLREEGLRTLPSEMQPSFLKRLQALDDSELDIPTLFELYELEKRRTMPYSGGIFGR

ORF TcrORF_05212

MSPSHLLDLALLLTAFAAVDSLKVKLREEGLRTLPSEMQPSFLKRLQALDDSELDIPTLFELYELEKRRTMPYSGGIFGR

**Npp 34**

WFU TcrWFU_03513

MRATCISILTLFYFVNSLRLHDLEGPADPAVSSALPVGEPEDDTDFPLPSRYRLAKRYPLLSDFDEEMMIGNPYWAKEISRRSPQFAWRPHSRFGRR

ORF TcrORF_10528

MRATCISILTLFYFVNSLRLHDLEGPADPAVSSALPVGEPEDDTDFPLPSRYRLAKRYPLLSDFDEEMMIGNPYWAKEISRRSPQFAWRPHSRFGRR

**Npp 35**

WFU TcrWFU_02815

MKNVSRYLMSQFSLRLILCCVVLAFFTNAVYGAPNGFERPVQIDDTPEELSLRDYLNLVRLKALKEEMDDGGYGEEESFEKRARFRPRLGKRSYNFRARLG

ORF TcrORF_06522

MKNVSRYLMSQFSLRLILCCVVLAFFTNAVYGAPNGFERPVQIDDTPEELSLRDYLNLVRLKALKEEMDDGGYGEEESFEKRARFRPRLGKRSYNFRARLG

**Npp 36**

WFU TcrWFU_04467 MCSLLQRKFYAVVLVTMFLVCFTASAPKPSQIESKLMKMEKLRDLLDMMEEEEISDALTVHPSYSKRWFPVKQYRGGLVAV

ORF TcrORF_05256 MCSLLQRKFYAVVLVTMFLVCFTASAPKPSQIESKLMKMEKLRDLLDMMEEEEISDALTVHPSYSKRWFPVKQYRGGLVAV

**Npp 37 (trimmed)**

WFU TcrWFU_00951

MHGFRPTLLLMAFVCVIAFVAMPAGSAGVYDATEPTFENDEVLEIPYRFIKRWTDFKKRSIPRRTLLPHFE

ORF TcrORF_07046

MHGFRPTLLLMAFVCVIAFVAMPAGSAGVYDATEPTFENDEVLEIPYRFIKRWTDFKKRSIPRRTLLPHFE

**Npp 40**

WFU TcrWFU_04102

MNPLSPIFLAFVAMTVITSTGAYPHWSVYDSLDTYGDYFNDVDRQYGQLQKRFLLGLGLTKNTPGKEIFLTSAASKKPYHRQ

ORF TcrORF_04940

MNPLSPIFLAFVAMTVITSTGAYPHWSVYDSLDTYGDYFNDVDRQYGQLQKRFLLGLGLTKNTPGKEIFLTSAASKKPYHRQ

**Npp 41 (trimmed)**

WFU TcrWFU_01638

MRALVLTLGLVMTHLATTTAVAVAVPSTLRSSISTEDSNAILPVLEPLEPMDMELHYLRQLQDMRNRVNAHRTSKRFFCNPYGCI

ORF TcrORF_08976

MRALVLTLGLVMTHLATTTAVAVAVPSTLRSSISTEDSNAILPVLEPLEPMDMELHYLRQLQDMRNRVNAHRTSKRFFCNPYGCI

**Npp 42**

WFU TcrWFU_10283

MGKLGMPFLVTLTLALLLTTRGGYCTRLQNAPPVMLTTAGLLASSPSQPPPDTYDFAPSIDDYWDEVHPPVLRKRPWALPDPLHCCLLRLSCCRRRGSSNYS

ORF TcrORF_02794

MGKLGMPFLVTLTLALLLTTRGGYCTRLQNAPPVMLTTAGLLASSPSQPPPDTYDFAPSIDDYWDEVHPPVLRKRPWALPDPLHCCLLRLSCCRRRGSSNYS

**Npp 43 (trimmed)**

WFU TcrWFU_08683

MPFDVRMLAIMALTASLFLTSASPMVTEKCFSSDKAMINYLLKKHALCYENSLDPDCLQLDIDKKATLAYFKRSLRSSELLPRDRRASLAYF

ORF TcrORF_02069

MPFDVRMLAIMALTASLFLTSASPMVTEKCFSSDKAMINYLLKKHALCYENSLDPDCLQLDIDKKATLAYFKRSLRSSELLPRDRRASLAYF

**Npp 44**

WFU TcrWFU_05513

MRMLTCLGLPLRLVIFITALGVITCSPWGLRDQEAGGRGDLDPLWLTRLLERQEPYDADLAMPMEPIDFGGRLKRTYARPAIRMG

ORF TcrORF_04021

MRMLTCLGLPLRLVIFITALGVITCSPWGLRDQEAGGRGDLDPLWLTRLLERQEPYDADLAMPMEPIDFGGRLKRTYARPAIRMG

**Neuropeptide prohorme 4**

WFU MHLLLFFIILLINQFSRGQYSVEGLRQQHNSNFDVDKEESDEPTGAAEMKEAVPVGNGCPVTAPWPCQQYSFCLSFAFVCDGEIDCPDGYDENPRLCVAKNRPAVALLEGFIRKYRDWLVPKYLGDGEPKFIAYNLAISQNIEDYRKNMQLTDEQFHNLARLLDEVAKGRQMGLLMLGMPLQSWSEVYIVLRPVAKGLLNASPVLHP

ORF MHLLLFFIILLINQFSRGQYSVEGLRQQHNSNFDVDKEESDEPTGAAEMKEAVPVGNGCPVTAPWPCQQYSFCLSFAFVCDGEIDCPDGYDENPRLCVAKNRPAVALLEGFIRKYRDWLVPKYLGDGEPKFIAYNLAISQNIEDYRKNMQLTDEQFHNLARLLDEVAKGRQMGLLMLGMPLQSWSEVYIVLRPVAKGLLNASPVLHP
